# Case of Paraplegia Successfully Treated by the Ergot of Rye

**Published:** 1852-02

**Authors:** 


*Case of Paraplegia Successfully Treated by the Ergot of Rye.*—Dr. Gerard, chief physician of the Hotel Dieu, of Marseilles, has published in the "Bulletin de Therapeutique," three cases, where from ten to forty grains of the ergot of rye have cured paraplegia of the inferior extremities. In the first case the paraplegia had lasted four years, in a man of thirty-nine; the ergot was administered in the dose of ten grains per diem, and gradually increased to forty. In three months the man was quite well. The second case refers to a man of twenty-nine, whose affection was caused by intemperance; he was cured in two months by the same means. The third patient, aged twenty-three, had suffered both from paraplegia and anæsthesia after having been much exposed to dampness in Algeria. The cure by the ergot was effected in three months.—*Ibid.*

